# Genome-Wide Identification, 3D Modeling, Expression and Enzymatic Activity Analysis of Cell Wall Invertase Gene Family from Cassava (*Manihot esculenta* Crantz)

**DOI:** 10.3390/ijms15057313

**Published:** 2014-04-28

**Authors:** Yuan Yao, Meng-Ting Geng, Xiao-Hui Wu, Jiao Liu, Rui-Mei Li, Xin-Wen Hu, Jian-Chun Guo

**Affiliations:** 1 Key Laboratory of Biology and Genetic Resources of Tropical Crops, Ministry of Agriculture, Institute of Tropical Bioscience and Biotechnology, Chinese Academy of Tropical Agricultural Sciences, Haikou 571101, China; E-Mails: yaofaraway1@163.com (Y.Y.); mengtinggeng8908@163.com (M.-T.G.); liujiao@itbb.org.cn (J.L.); liruimei@itbb.org.cn (R.-M.L.); 2 Agricultural College of Hainan University, Haikou 571104, China; E-Mail: ficz@163.com

**Keywords:** molecular cloning, cell wall invertase, cassava, gene expression analysis, 3D modeling, enzyme activities

## Abstract

The cell wall invertases play a crucial role on the sucrose metabolism in plant source and sink organs. In this research, six cell wall invertase genes (*MeCWINV1-6*) were cloned from cassava. All the MeCWINVs contain a putative signal peptide with a predicted extracellular location. The overall predicted structures of the MeCWINV1-6 are similar to AtcwINV1. Their *N*-terminus domain forms a β-propeller module and three conserved sequence domains (NDPNG, RDP and WECP(V)D), in which the catalytic residues are situated in these domains; while the *C*-terminus domain consists of a β-sandwich module. The predicted structure of Pro residue from the WECPD (MeCWINV1, 2, 5, and 6), and Val residue from the WECVD (MeCWINV3 and 4) are different. The activity of *MeCWINV1* and *3* were higher than other *MeCWINVs* in leaves and tubers, which suggested that sucrose was mainly catalyzed by the MeCWINV1 and 3 in the apoplastic space of cassava source and sink organs. The transcriptional levels of all the *MeCWINVs* and their enzymatic activity were lower in tubers than in leaves at all the stages during the cassava tuber development. It suggested that the major role of the MeCWINVs was on the regulation of carbon exportation from source leaves, and the ratio of sucrose to hexose in the apoplasts; the role of these enzymes on the sucrose unloading to tuber was weaker.

## 1. Introduction

Cassava (*Manihot esculenta* Crantz) is a widely cultivated tuber crop due to its high starch content and is a staple food for more than 500 million people in tropical and subtropical regions of Africa, Asia and Latin America. The rate of CO_2_ fixation and sucrose synthesis in cassava leaves (source organ) is highest among all C3 plants; however, its tuber roots (sink organ) accumulate a small part of carbohydrates and rarely reach the yield potentials in the field [1]. This means that cassava has high source strength, while its sink strength is lower. Targeted modification of enzymes regulating the source–sink relationship can increase both tuber root number and total tuber root biomass of cassava [1]. Therefore, it is meaningful to research on the key enzymes that relate to cassava sucrose metabolism, starch synthesis and the regulation of the source–sink relationship in cassava.

In higher plants, the invertases irreversibly hydrolyze sucrose into glucose and fructose, and they are the key enzymes in sucrose metabolism [2,3]. There are three types of invertase isoenzymes (vacuolar invertase, cell wall invertase and alkaline/neutral invertase) in higher plants, which are distinguished based on their subcellular localization, solubility, optimum pH and isoelectric point [4]. The vacuolar and cell wall invertases have similar enzymatic and biochemical properties: they have an acidic pH optimum, thus are labeled as acid invertases. The acid invertases are also able to cleave stachyose and raffinose, but with significantly reduced cleavage efficiency [4]. The acid invertases are glycoproteins and have three conserved sequence motifs: β-fructofuranosidase motif (NDPNG(A)), RDP and WECP(V)D motifs [4–6]. The Asp locating in the NDPNG(A) and RDP, and the Glu and Cys locating in the WECP(V)D are catalytic residues in the cell wall invertase (AtcwINV1) of *Aribidopsis thaliana* and the vacuolar invertase (Bobfruct3) of *Bambusa oldhamii* [5,7]. All the cell wall invertases contain a Pro residue in the WECPD motif, whereas, in all the vacuolar invertases, the Val residue instead of the Pro residue in WECPD motif, which consists of WECVD [4,6]. According to the research on the acid invertases in *Chenopodium rubrum*, the difference of the residue in WECPD determines the cell wall invertases with higher acidic pH-optimum and higher cleavage rate of raffinose than the vacuolar invertases with WECVD [8]. The vacuolar invertases are thought to function in control of sugar composition in fruits and storage organs [9], osmoregulation and cell enlargement [10], and in response to drought stress [4,11]; while the proposed functions of the cell wall invertases include the regulation of sucrose partitioning [12], response to wounding and pathogen infection [13], regulation of seed, and pollen development [14]. In the source organs (leaves) of the tomato, the cell wall invertases restrict carbon to export from source leaves and regulate the ratio of sucrose to hexose in apoplasts [15]; while the cell wall invertases play an important role in apoplastic cleavage of sucrose throughout the sink organ (seed) development of maize [16]. This means that the cell wall invertases regulate the sucrose metabolism in both source and sink organs in higher plants. In cassava, the genes and their functions of the cell wall invertases are not reported. In the present study, six cell invertase genes from cassava were cloned according to the released sequence of the cassava genome. The evolutionary relationships, exon-intron structure, motif distribution, subcellular localization prediction and protein 3D structure of the six family genes were investigated. To elucidate the possible roles of the cell wall invertase genes, the spatial and tissue differential expressions and enzymatic activity of these genes were investigated in source and sink organs during plant and tuber root development. These results will be helpful to further the understanding of the possible roles of cell wall invertase in sucrose metabolism in cassava.

## 2. Results

### 2.1. Identification and Characterization of the MeCWINV Genes in Cassava

**Figure 1.**
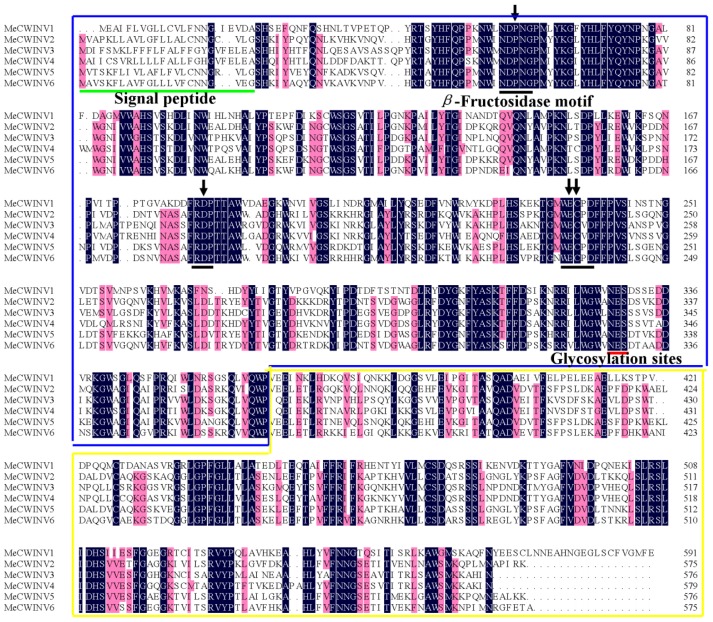
Protein alignment and domain structure of the *MeCWINVs*. Three conserved sequence domains—NDPNG (β-fructosidase motif), RDP and WECP(V)D—which contain the predicted active sites of the enzyme, are shown in black lines. Red lines indicate the predicted glycosylation sites (NES). Green line indicates the putative signal peptide. Four arrows show the amino acids, which correspond with the predicted active residues of invertase. The *N*-terminus domain sequence is shown in the blue box, and the *C*-terminus domain sequence is shown in the yellow box.

In order to identify cell wall invertase genes in cassava, CWINV protein sequences from *A. thaliana* and *O. sativa* were used as queries in a BLAST search with the publicly available cassava sequence database (http://www.phytozome.net/cassava) [17]; and six putative cell wall invertase genes, named *MeCWINV1-6*, are found in the cassava genome. The *MeCWINVs* are located in five different scaffolds in cassava genome database. *MeCWINV2* and *5* are collinearly arranged at scaffold 06550, which indicates that a chromosomal duplication event gave rise to these two genes. Full-length cDNAs of the six *MeCWINV* genes were cloned by RT-PCR using gene-special primers. The 3′ region of *MeCWINV1* sequence was obtained using 3′ RACE technology, due to this region being missed by BLAST search. The cDNA and the deduced amino acid sequences of the *MeCWINVs* described in this study were deposited in GenBank under the following accession numbers: *MeCWINV1* (JQ339929), *MeCWINV2* (JX291160), *MeCWINV3* (JN801147), *MeCWINV4* (JQ792172), *MeCWINV5* (JX291159), *MeCWINV6* (JQ339930). The ORF length of the six genes is between 1728 and 1779 bp, and their deduced amino acids range from 575 to 592 (Table 1). Alignment analysis of the deduced amino acids shows that the MeCWINVs share 48.25%–80.38% identities among all the six genes (Table S1). All the identified MeCWINVs have three conserved sequence domains—NDPNG (β-fructosidase motif), RDP and WECP(V)D—which contain the predicted active sites of the enzyme, and the predicted conserved glycosylation sites (NES) (Figure 1). Analysis with the Signal P 4.1 Server shows that all the MeCWINVs contain a putative signal peptide with a predicted cleavage site between amino acids 22 and 26 (Figure 1). The subcellular localization of the MeCWINV proteins using TargetP 1.1 programs shows that they are putatively located at extracellular space, which is consistent with the extracellular location of all cell wall invertases in other plants. These characteristic features suggest that they are the members of cell wall invertase gene family.

### 2.2. Gene Structure Analysis of the MeCWINV Genes

**Figure 2.**
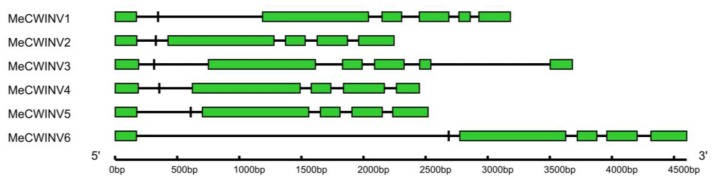
Exon-intron structure of the six cell wall invertase genes in cassava. Introns are shown as black lines, exons are shown as green boxes, and the mini exon is shown in the black box.

The gene structures of the *MeCWINV* genes were determined by aligning the cDNA sequences with the genomic sequence from the cassava genome database (http://www.phytozome.net/cassava) [17]. Based on their exon-intron structures, the *MeCWINV* isoforms are classified into two groups. *MeCWINV2*, *4*, *5* and *6* have six exons, among which the gene structures of *MeCWINV2*, *4* and *5* are similar; while *MeCWINV1* and *3* have seven exons. The second exon in all the *MeCWINVs* encodes three amino acids (DPN) and is included in the β-fructosidase motif, which is a mini exon the smallest one known in plants (Figure 2).

### 2.3. Phylogenetic Analysis of the MeCWINV Genes

**Figure 3.**
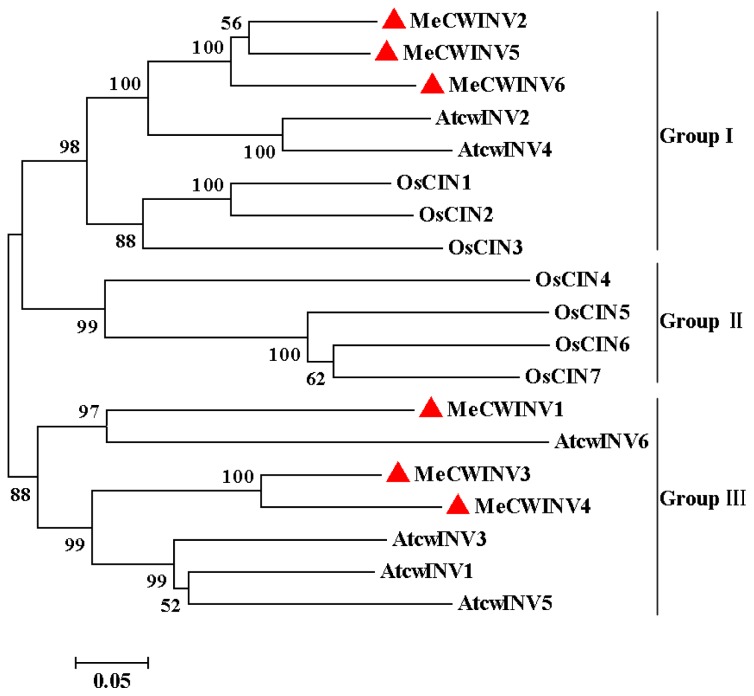
Relationship of the MeCWINVs with *O. sativa* and *A. thaliana* cell wall invertases. The tree was constructed based on the amino acid sequences of the cell wall invertases from cassava (MeCWINV1-6), *O. sativa* (OsCIN1-7) and *A. thaliana* (AtcwINV1-6). Alignment analysis was performed with ClustalW and cladistic using MEGA5. The tree was constructed using neighbor-joining algorithm.

The phylogenetic relationship of the MeCWINVs in cassava was compared with the cell wall invertases from *O. sativa* (OsCIN1-7) and *A. thaliana* (AtcwINV1-6), based on their amino acid sequences using the MEGA5 program. All the cell wall invertases used in this analysis are classified to three major sub-families and named as Group I to III, in which MeCWINV2, 5, 6, AtcwINV2, 4, and OsCIN1-3 are clustered to the Group I, while all the Group II members are from *O. sativa* (OsCIN4-7); MeCWINV1, 3, 4, and AtcwINV1, 3, 5, 6 are clustered to the Group III (Figure 3). In the Group I, MeCWINV2, 5, 6 are clustered to one branch, AtcwINV2, 4 are clustered to another branch, and they share 77.59% amino acid identity between the two branches. In the Group III, MeCWINV1 has a close relationship to AtcwINV6, and they share 50.25% amino acid identity; whereas MeCWINV3 and 4 are clustered with AtcwINV1, 3, 5, and they share 72.03% amino acid identity.

### 2.4. Structure Prediction and Homology Modeling of the MeCWINV Genes

**Figure 4.**
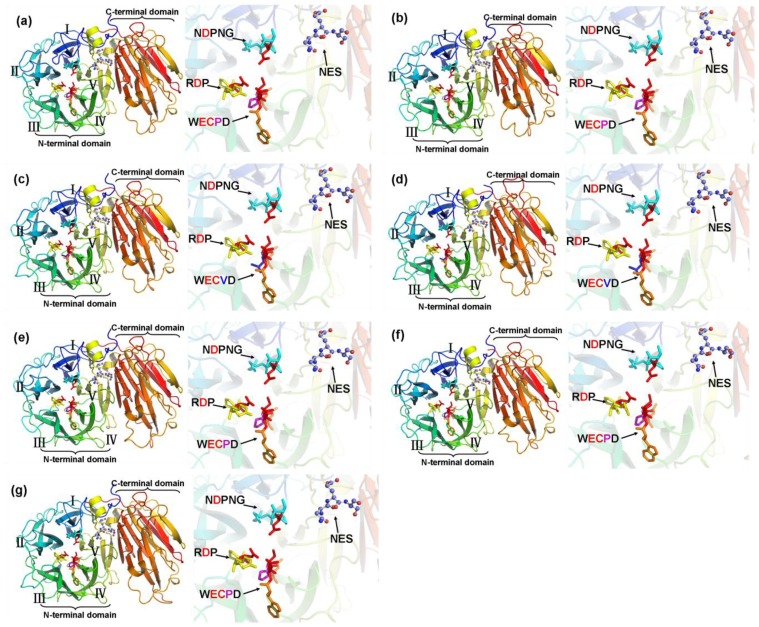
The cartoon representation and active sites of the predicted 3D structure model of MeVINV1-6 and AtcwINV1. (**a**–**f**) are the predicted 3D structure model of MeVINV1-6, respectively; (**g**) is the 3D structure model of AtcwINV1. Roman numerals (I–V) show the five blades of the β-propeller module, respectively. The graphics at the right side are the close-up views of the active sites. The conserved sequence domains (NDPNG, RDP, and WECP(V)D) are shown in sticks, and the red color sticks indicate the predicted catalytic residues. Stick and ball show the predicted glycosylation sites.

**Figure 5.**
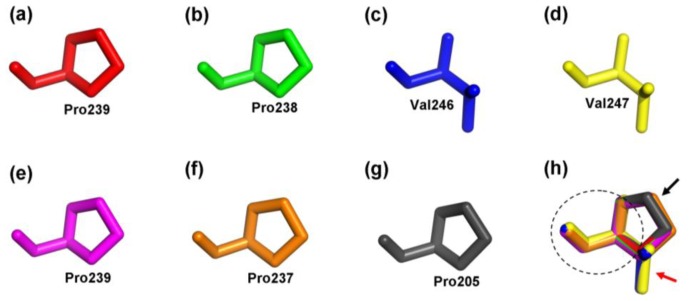
The close-up view of the predicted Pro and Val residue structure in WECP(V)D domain. (**a**–**f**) The predicted structure of Pro and Val residues within MeCWINV1-6, respectively; (**g**) The structure of Pro residue within AtcwINV1; (**h**) The comparison of the predicted Pro and Val residue structures of MeCWINV1-6, and Pro residue structure of AtcwINV1 in WECP(V)D domain. Dashed region shows the part of Pro and Val residue structures with the same orientation. Black arrow shows the cyclic structure of Pro residue. Red arrow shows a part of Val residue.

In order to obtain a reasonable theoretical structure of the MeCWINVs, protein homology modeling was performed using a Swiss model server. To predict the 3D structure of the MeCWINVs, a 3D structure at 2.15 Å of *A. thaliana* cell-wall invertase 1 (AtcwINV1; gene accession code: At3g13790; PDB id: 2AC1.1) was used as a template, which shares 58.82%, 60.73%, 68.49%, 65.41%, 59.47% and 57.93% sequence identity with MeCWINV1 to 6, respectively. The predicted 3D model of MeCWINV1-6 was validated with the QMEAN server for model quality estimation. The total QMEAN-score (estimated model reliability between 0 and 1) of the predicted 3D models for MeCWINV1 to 6 are 0.711 (Z-score: −0.63), 0.724 (Z-score: −0.48), 0.771 (Z-score: 0.07), 0.758 (Z-score: −0.07), 0.709 (Z-score: −0.67), and 0.736 (Z-score: −0.34), respectively. It indicates that all the sequences of MeCWINV1-6 match the homologous templates well on the server, so the models are reliable. The overall predicted structures of MeCWINV1-6 with substrate are similar to AtcwINV1. The conserved motifs NDPNG, RDP and NES within the MeCWINV1-6 and AtcwINV1, and the WECPD motif within the MeCWINV1, 2, 5, 6 and AtcwINV1 have the same orientation in the predicted 3D structure (Figure 4). The Pro residue in WECPD of MeCWINV1, 2, 5, 6 and AtcwINV1 have the similar predicted 3D structure (Figure 5a,b,e–g); and also the similar predicted 3D structure is found in the Val residue in WECVD of MeCWINV3 (Figure 5c,d); however, the Val instead of Pro residue forms different predicted 3D structure, which Pro residue forms a cyclic structure, and Val residue forms a open structure, but part of their structure orientation is similar (Figure 5h).

### 2.5. The Differential Expression Analysis of the MeCWINV Genes in Cassava Organs or Tissues

**Figure 6.**
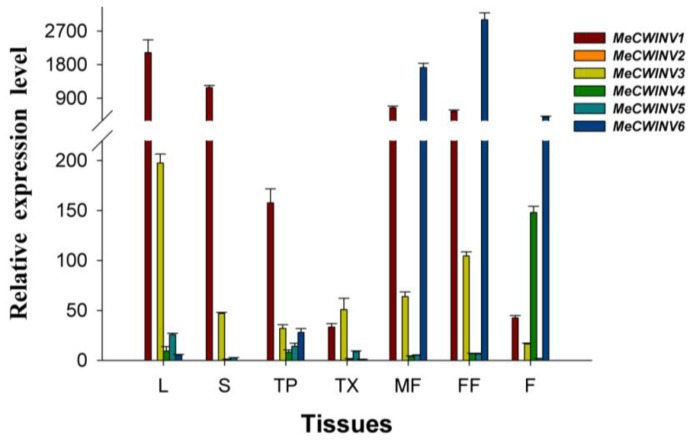
The differential expression of the *MeCWINV* genes in cassava organs or tissues. The amount of *MeCWINV* mRNA was normalized by tubulin mRNA. The expression of *MeCWINV4* in stems was used as a calibrator. Each value is the mean ± SE of three biological replicates (*n* = 3). Notes: L, Leaf, S, Stem, TP, Tuber phloem, TX, Tuber xylem, MF, Male flower, FF, Female flower, F, Fruits.

To investigate the tissue-specific expression patterns of the *MeCWINV* genes, total RNA was extracted and cDNA was synthesized separately from leaves, stems, tuber phloem, tuber xylem, male and female flowers and fruits of cassava for quantitative real-time PCR analysis. The result showed that the most highly expressed genes in leaves, stems and tuber were *MeCWINV1* and *3*; in male and female flowers were *MeCWINV1*, *3* and *6*; in fruits were *MeCWINV4* and *6*. Thus, *MeCWINV1*, *3* were active in most organs, except in fruits; *MeCWINV6* was active in reproductive organs; and *MeCWINV4* was more active in fruits (Figure 6). *MeCWINV2* was specifically expressed in male flowers with low level. The expression of *MeCWINV5* was comparably low, but it could be found in all organs. Remarkably, almost all of the *MeCWINVs* were weakly expressed in tuber xylem when the plant was at 90 days after planting (Figure 6).

### 2.6. The Differential Expression of the MeCWINV Genes during Cassava Tuber Root Development

The differential expression of the *MeCWINV* genes was examined in leaves, tuber xylem and tuber phloem of the cassava plant using qRT-PCR at 90, 135, 180, 225 and 270 days after planting, during tuber root development. The cassava plant initially develops tuber roots at 90 days, expands tuber roots with starch accumulation at 135 and 180 days, and tuber roots mature at 225–270 days. The expression of *MeCWINV6* in leaves at 90 days was used as a calibrator to compare with other genes in leaves, tuber xylem and tuber phloem for all map-making.

**Figure 7.**
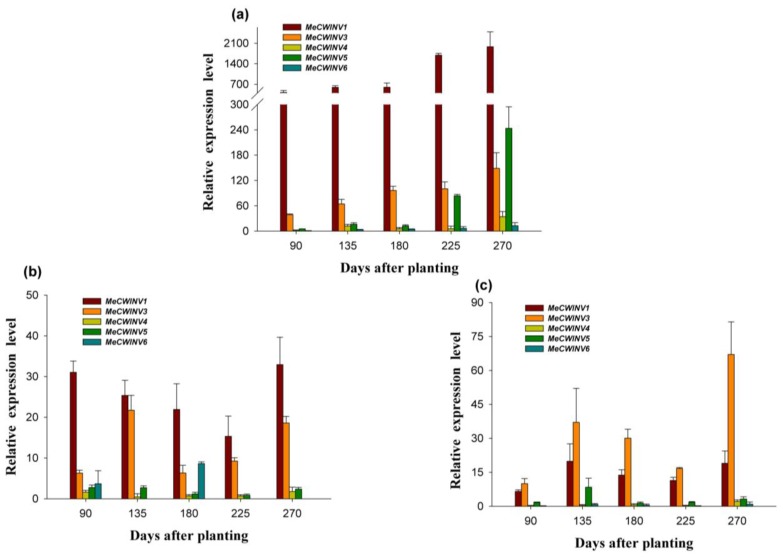
The differential expression analysis of the *MeCWINV* genes in cassava leaves (**a**); tuber phloem (**b**) and tuber xylem (**c**) during tuber developmental stages. The differential expression analyses were at 90, 135, 180, 225 and 270 days after planting. Each value is the mean ± SE of three biological replicates. The amount of *MeCWINV* mRNA was normalized by tubulin mRNA.

In leaves, *MeCWINV1* and *MeCWINV3* were expressed at all stages, but the relative expression level of *MeCWINV3* was lower than *MeCWINV1* (Figure 7a). The activity of the both genes was increased with the tuber root maturity (Figure 7a). *MeCWINV5* was active at the mature tuber stage. The expression of *MeCWINV4* and *6* was low at almost all stages, and no expression was found in *MeCWINV2* (Figure 7a).

In tuber phloem, the highly most expressed genes at all stages were *MeCWINV1* and *3*; the highest expression of *MeCWINV1* was at the later mature tuber stage of 270 days, followed by the tuber root initial stage (90 days) and early expanding stage (135 days), while the highest expression of *MeCWINV3* was at the tuber root early expanding stage (135 days), followed by the mature tuber stage, and it was lower at the other stages. The relative mRNA level of *MeCWINV1* was higher than that of *MeCWINV3* at all stages. The activity of *MeCWINV6* was found at the later expanding tuber stage of 180 days, but it was lower at other stages. *MeCWINV4*, and *5* maintained low expression levels at all stages, and no expression was found in *MeCWINV2* (Figure 7b). The relative expression levels of all the *MeCWINVs* in tuber phloem were lower than in leaves (Figure 7a,b).

In tuber xylem, the analysis of *MeCWINVs* expression revealed that *MeCWINV1* and *3* maintained higher expression levels at all tuber development stages; the highest expression of *MeCWINV3* was at the later mature tuber stage of 270 days, and the relative mRNA level of *MeCWINV3* was higher than that of *MeCWINV1* at all stages (Figure 7c). The activity of *MeCWINV5* was found at early expanding stage (135 days), and it was very low at other stages. *MeCWINV4* and *6* maintained low expression at all the stages, and no expression was detected in *MeCWINV2* at all stages (Figure 7c). The relative expression levels of all *MeCWINVs* in tuber xylem were lower than in leaves (Figure 7a,c).

### 2.7. The Activity of Cell Wall Invertase in Cassava Leaves and Tubers during Tuber Root Development

**Figure 8.**
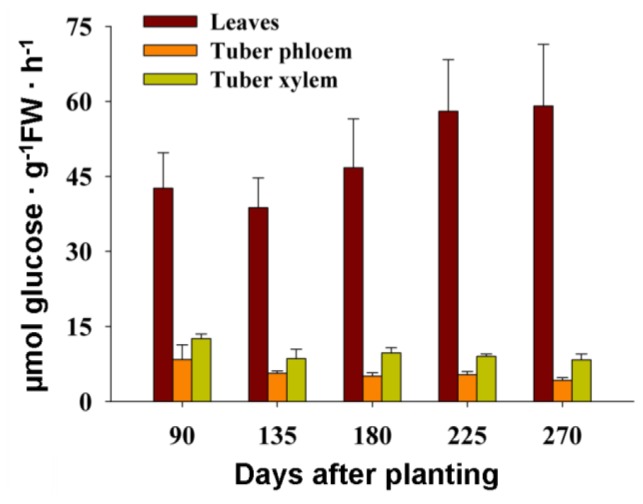
The activity analysis of the cell wall invertase in cassava leaves, tuber phloem and tuber xylem during tuber developmental stages. Activities were expressed as μmol glucose synthesized g^−1^·FW·h^−1^. Each value is the mean ± SE of three biological replicates.

In order to study the role of the cell wall invertases MeCWINVs in source and sink organs during cassava tuber root development, the activity of the cell wall invertase in cassava leaves, tuber phloem and xylem was measured at 90, 135, 180, 225 and 270 days after planting. The result showed that the activity of the cell wall invertase in leaves was lower at tuber initial stage (90 days) and expanding stage (135, 180 days) than at mature tuber stage (225, 270 days). In tuber phloem and xylem, the activity of the cell wall invertase was higher at tuber initial stage than at the other stages of 135, 180, 225 and 270 days. The activity of the cell wall invertase in leaves was always higher than in tuber phloem and xylem at all stages, and in tuber phloem was always lower than in leaves and tuber xylem (Figure 8).

## 3. Discussion

### 3.1. Identification and Characterization of the MeCWINV Genes

In this study, we identified six cell wall invertase genes from cassava genome. Researches have reported that the members of the cell wall invertase genes vary in plants, such as in *Lycopersicon esculentum*, there are four cell wall invertase members [2,13]; in *A. thaliana*, there are six members [18]; in *Populus trichocarpa*, they are five members [19]; in *O. sativa*, there are seven members [6]. Phylogenetic analysis of the cell wall invertases CWINVs from *M. esculenta*, *O. sativa* and *A. thaliana* shows that the MeCWINVs have a closer evolutionary relationship with the cell wall invertases from *A. thaliana* than that from *O. sativa* (Figure 3). Both *MeCWINV2* and *5* are collinearly arranged at scaffold 06550, which indicates that a chromosomal duplication event gave rise to these two genes. Similarly, in the potato genome, *InvGE* and *InvGF*, *InvCD111* and *InvCD141* are collinearly arranged on the chromosomes of 9 and 10, respectively [20]. This phenomenon also appears in the acid invertase gene family of *AtcwINV1* and *AtcwINV5* from *A. thaliana*, *PtCIN4* and *PtCIN5*, *PtCIN1* and *PtCIN2* from *P. trichocarpa*, *Lin5* and *Lin7*, *Lin6* and *Lin8* from *Solanum lycopersicum* [19,20]. The MeCWINVs are encoded by six or seven exons, and the second mini exon encoding DPN is a part of β-fructosidase motif NDPNG(A), which the exon-intron structure are consistent with all the reported cell wall invertases in other plants, such as in *P. trichocarpa* [19] and in *O. sativa* [6]. *MeCWINV2*, *5*, and *6* are classified to the Group I; they have six exons and share 74.70%–80.38% amino acid identity. *MeCWINV1*, *3*, and *4* are classified to the Group III, *MeCWINV1* and *3* have seven exons, while *MeCWINV4* has six exons; *MeCWINV3* and *4* share 77.93% amino acid identity. The events of intron loss are common in the evolutionary history of the cell wall invertase gene family of *O. sativa* and *A. thaliana* [21]. Therefore, we speculate that *MeCWINV4* has lost an intron in the evolutionary history. It has been reported that within the conserved WECP(V)D sequence domains of the acid invertases, all the cell wall invertases have a Pro residue, and all the vacuolar invertases possess a Val residue [4]; however, within the conserved WECP(V)D sequence domains of MeCWINV3 and 4, the Pro residue is replaced by Val residue. Goetz and Roitsch (1990) reported that the Pro residue in WECP(V)D sequence determines the cell wall invertases with lower acidic pH optimum, and more effective degradation to raffinose in comparison with the vacuolar invertases with a Val residue at this place [8]. Therefore, we speculate that MeCWINV3 and 4 might have higher pH optimum and lower effective degradation to raffinose than MeCWINV1, 2, 5, and 6. It was reported that the vacuolar invertase had evolved from the cell wall invertase during earlier plant evolutionary history [21]. Our finding in the conserved WECP(V)D might provide evidence for this hypothesis. However, *Physcomitrella patens* might lack true cell wall invertases, but has six acid invertases which have a close relationship to the vacuolar invertases in *A. thaliana* [22]. Thus, the evolutionary relationships between these two different acid invertases are unclear.

### 3.2. Protein Structural Features of the MeCWINVs in Cassava

The invertases are a member of glycosidase family GH32, and the first three-dimensional crystal structure of the invertase (β-Fructosidase) from *Thermotoga maritime* was reported by Alberto *et al.* (2004) [23]. The three-dimensional crystal structure of the cell-wall invertase 1 (AtcwINV1) from *A. thaliana* was first reported in plant invertase [24]. Our results show that the predicted three-dimensional structures of MeCWINV1-6 are similar to AtcwINV1, in which their *N*-terminus domain forms a five-bladed β-propeller module, and three conserved sequence domains (NDPNG, RDP and WECP(V)D) that contain catalytic residues in this module, while their *C*-terminus domain consists of a β-sandwich module. The conserved structures in the cell wall invertases ensure that the catalytic center is for sucrose degradation [5]. The structure of WECPD in MeCWINV1, 2, 5, 6, and AtcwINV1 is similar to the WECVD in MeCWINV3 and 4, but the Pro residue in WECPD forms different predicted 3D structure with Val residue in WECVD, which Pro residue forms a cyclic structure, and Val residue forms a open structure, but part of their structure orientation is similar.

### 3.3. Differential Expression and Enzymatic Activity Analysis of the MeCWINV Genes

The tissue-specific expression pattern of the *MeCWINV* genes could provide a basis for understanding their functions during cassava plant development. *MeCWINV1*, *3*, *6* were highly expressed in male and female flowers; *MeCWINV4* was highly expressed in fruits; *MeCWINV1*, *3*, were highly expressed in leaves; *MeCWINV2* was specifically expressed in male flowers. This suggests the highly expressed *MeCWINV* genes play an important role in those organs or tissues, respectively. It was reported that there are four cell wall invertases as *Lin5*, *6*, *7*, *8* in tomato, in which *Lin5* is active in ovaries and fruits, and *Lin7* is active in stamens and pollen; *Lin7* and *8* mainly express in vegetative organs (root and leaf) [25]. In the growing pollen tubes of *A. thaliana*, the cell wall invertase irreversibly hydrolyzed sucrose into monosaccharide, and then this was transported into pollen tubes by a H^+^/monosaccharide transporter [26,27]. Our results found that almost all the *MeCWINV* genes were weakly expressed in tuber xylem when the plant was at 90 days after planting (Figure 6). During the cassava tuber root development at 90–270 days after planting, the relative expression levels of the *MeCWINV* genes in leaves were higher than in tuber phloem and xylem (Figure 7). *MeCWINV1*, *3* were highly active in leaves at all stages of tuber root development, while *MeCWINV5* was only highly active in leaves at tuber maturity stage (225, 270 days) (Figure 7a). The enzymatic activity of the total cell wall invertases was much higher in leaves than in tuber phloem and xylem, which was consistent with the transcription levels of cell wall invertases during cassava tuber root development (Figure 8). Noticeably, the gene expression level and enzymatic activity of cassava cell wall invertase in source organs (leaves) was lower at tuber initial stage (90 days) and expanding stage (135, 180 days), and it was higher at tuber maturity stage (225, 270 days). In the source organs (leaves) of tomato, the cell wall invertase functions to restrict carbon exporting out from source leaves, which regulates the ratio of sucrose to hexose in the apoplast [15]. Our results indicate that low efficiency of sucrose hydrolysis in leaves occurs at the initial stage of tuber, which may benefit the export of carbon from source organs (leaves) to sink organs (tuber).

In higher plants, there are three different routes for sucrose uptake into sink organs: (a) import by sucrose transporter; (b) cleavage of sucrose by cell wall invertase; subsequently carrying by hexose transporter or (c) uptake by plasmodesmata. The sucrose phloem unloading pathway found in the maize seeds [28], in carrot tap root [29], and in tomato fruit [25] is mainly via the cell wall invertase and hexose transporters. Sucrose transporter and cell wall invertase play a crucial role in the early potato tuber development, while most of the sucrose is unloaded via plasmodesmata during later tuber growth [30,31]. However, the process of sucrose uptake into cassava tuber is unclear. *MeCWINV1* and *3* were highly active in both leaves and tuber, which suggests that the decomposition of sucrose in the apoplastic space was mainly catalyzed by the MeCWINV1 and 3 in cassava source and sink organs. However, the enzyme activity was not consistent with the pattern of the *MeCWINV* gene expression in tubers. The activity of CWINV is tightly regulated at both the transcriptional and the posttranscriptional levels [32]. At the posttranslational level, CWINV activity can be regulated by invertase proteins [33]. Therefore, we speculate that invertase inhibitor might regulate the MeCWINV activity. The transcriptional level of *MeCWINVs* and their enzymatic activity in tubers (tuber phloem and xylem) were much lower than in leaves at all the stages of tuber development, which suggests that the major role of cassava cell wall invertases was in the regulation of carbon exportation from source leaves, and the sucrose to hexose ratio in the apoplast. The role of these enzymes on the sucrose unloading to tuber was weaker.

## 4. Experimental Section

### 4.1. Plant Materials

Cassava cultivar SC8 (*Manihot esculenta* Crantz no SC8) obtained from the Tropical Crops Genetic Resource Institute (TCGRI, Danzhou, China), Chinese Academy of Tropical Agricultural Sciences (CATAS, Haikou, China) was planted in field under natural conditions with the average temperature of 23.8 °C. In order to find out the different role of MeCWINVs in tuber phloem (be responsible for sucrose phloem unloading and storage small amount of starch) and tuber xylem (main storage tissue) during tuber development, tuber phloem and tuber xylem are separated by cutting tuber phloem. For gene cloning and differential expression analysis in tissues and organs, the plant materials were collected as follows: the leaves, stems, tuber phloem and tuber xylem were collected 90 days after planting; the male and female flowers were collected 200 days after planting; and the fruits were collected 225 days after planting. For differential expression analysis of these genes in source and sink organs during tuber root development, the plant materials were collected as follows: the leaves, tuber phloem and tuber xylem were collected at 90, 135, 180, 225 and 270 days after planting. Three biological samples (different plants) were collected for analyses. We have collected two cassava tubers from individual plant and slit the tuber phloem and tuber xylem in the middle part. All materials were immediately frozen in liquid nitrogen and stored at −80 °C for RNA isolation.

### 4.2. Cloning of the Full-Length MeCWINV Genes

Total RNA was extracted from cassava tissues using RNAplant Plus reagent (TianGen, Beijing, China) following the manufacturer’s instructions. Total RNA was reversely transcribed using the RNA PCR Kit (AMV) Ver.3.0 and Oligo dT-Adaptor Primer (TaKaRa, Dalian, China). Full-length cDNAs of the *MeCWINVs* genes were isolated by RT–PCR, using a set of gene-specific primers (Table 2) which were designed based on BLAST analysis of the cassava genome database (http://www.phytozome.net/cassava) [17], using the published sequences of the cell wall invertase gene *OsCIN1-7* in *Oryza sativa* and *AtcwINV1-6* in *Arabidopsis thaliana*. The 3′ region of the *MeCWINV1* sequence was obtained using 3′ RACE technology due to this region being missing by BLAST search. For *MeCWINV1* full-length amplification, RT-PCR was carried out using a forward primer, which is specific to the sequence taken from the BLAST search and a reverse primer, which is designed based on sequence obtained from 5′ RACE PCR product. All the PCR products were ligated into the pMD-18 T vector (Takara, Dalian, China) and were sequenced (Shanghai Sangon Biological Engineering Technology and Services CO., Ltd, Shanghai, China).

### 4.3. Sequence Alignment and Phylogenetic Analysis

Multiple-sequence alignments of the *MeCWINV* genes, and their deduced amino acids were analyzed by DNAman 6.0 software (Lynnon Biosoft, Quebec, QC, Canada). The evolutionary relationship of MeCWINVs was analyzed using the Neighbor-Joining method of the MEGA5 program according to the deduced amino acids with 1000 bootstrap replicates. The evolutionary distances were computed using the Poisson correction method. The sequences used for phylogenetic analysis were cell wall invertases in *Oryza sativa* (OsCIN1-7) and *Arabidopsis thaliana* (AtcwINV1-6).

### 4.4. Signal Peptide and Subcellular Localization Prediction

The signal peptide sequence and putative cleavage site of MeCWINVs were predicted using the SignalP program (http://www.cbs.dtu.dk/services/SignalP/) [34]. MeCWINVs amino acid sequences were subjected to computer analysis for subcellular localizations using web tools, TargetP 1.1 program (http://www.cbs.dtu.dk/services/TargetP/) [35].

### 4.5. Exon-Intron Structure Analysis

The exon-intron structure of each *MeCWINV* gene was determined by aligning the cDNA sequence of the cell wall invertase with the genomic sequence in the cassava genome database (http://www.phytozome.net/cassava) [17]. The gene schematic structure was drawn by the Gene Structure Display Server (http://gsds.cbi.pku.edu.cn/index.php) [36].

### 4.6. Homology Modeling and Structure Prediction

Protein sequences of plant cell wall invertases MeCWINV1-6 were submitted to the Swiss-Model server (http://swissmodel.expasy.org) [37] to perform sequence analysis, and the cell-wall invertase 1 (AtcwINV1; gene accession code: At3g13790; PDB id: 2AC1.1) from *Arabidopsis thaliana* was applied as a template. The catalytic and enzymatically important residues of MeCWINVs were displayed using the Pymol software (Delino Scientific, San Carlos, CA, USA).

### 4.7. Real-Time RT-PCR Analysis

Total RNA was extracted from each biological sample using RNAplant Plus reagent (TianGen, Beijing, China) according to the protocol of the manufacturer. Reverse transcription was carried out with the PrimeScript™ RT reagent Kit with gDNA Eraser (Perfect Real Time) (TaKaRa, Dalian, China) according to the manufacturer’s protocol. The relative mRNA expression of *MeCWINVs* was analyzed by quantitative real-time RT-PCR (qRT-PCR) using the qRT-PCR primers given in Table 3. We designed qPCR primers for each *MeCWINV* gene on the region of low sequence similarity to the other genes, and the amplified fragments were sequenced to ensure their specificity. The reactions were performed in a 384-well plate in a volume of 10 μL containing 5 μL 2× SYBR^®^ Premix Ex Taq II (Tli RNaseH Plus), 0.2 μL ROX Reference Dye (50×), 0.2 μL forward and reverse primers (10 μM), 0.4 μL H_2_O, 4 μL template cDNA (SYBR green reagents were supplied by Takara, Dalian, China). The Applied Biosystems HT7900 apparatus was programmed with the following amplification protocol: 1 min at 95 °C for one cycle, followed by 45 cycles of PCR (5 s at 95 °C, 30 s at 60 °C). The amplification program was followed by a melting curve analysis consisting of 95 °C for 15 s, 60 °C for 15 s, and 95 °C for 15 s. The temperature ramp rate was set to 100% for all steps except the final ramp between 60 and 95 °C, which was set to 5%. Three technical replicates for each biological sample were analyzed. The relative expression was calculated according to the 2^−ΔΔ^*^C^*^t^ method [38]; as an internal control, cassava tubulin gene mRNA was amplified (Tubulin-F: 5′ GTGGAGGAACTGGTTCTGGA 3′ and Tubulin-R: 5′ TGCACTCATCTGCATTCTCC 3′) in an identical manner.

### 4.8. Activity Analysis of the Cell Wall Invertase

The pellet mix procedure was used to assess the activities of cell wall invertase [39]. About 0.8 g of each biological sample was homogenized in an ice-cold buffer at 1:4 (*w*/*v*) ratio containing 62.5 mM Tris-HCl (pH 6.8), 2 mM EDTA (pH 8.0), 10% (*v*/*v*) glycerol (85%) and 10 mM β-mercaptoethanol [40]. After centrifugation at 12,000× *g* for 30 min at 4 °C, we removed the supernatant, washed the pellet three times with extraction buffer and used it for activity assay of cell wall bound invertases after final resuspension in at 1:4 (*w*/*v*) ratio of extraction buffer. The activities were assayed in a final volume of 0.5 mL, containing 125 mM Na-acetate buffer (pH 4.5), 100 mM sucrose and 0.035 mL of the pellet mix, and started the reactions after adding enzyme extract followed by incubation at 37 °C for 30 min. Reactions were stopped at boiling water for 1 min and sucrose was omitted from the reaction medium for blanks. Three technical replicates for each biological sample were analyzed. Glucose production of each stopped reaction mixture was measured by the oxidase-peroxidase (GOD-POD) method.

## 5. Conclusions

In conclusion, six cell wall invertase genes (*MeCWINV1-6*) were cloned from cassava. All MeCWINVs contains a signal peptide with a predicted extracellular location. Their *N*-terminus domain forms a β-propeller module, and conserved sequence domains (NDPNG, RDP and WECP(V)D), which contain catalytic residues, are situated in this domain, while the *C*-terminus domain consists of a β-sandwich module. Sucrose was mainly catalyzed by the MeCWINV1 and 3 in the apoplastic space of cassava source and sink organs. The transcriptional level of *MeCWINVs* and their enzymatic activity in tubers were lower than in leaves at all stages during the cassava tuber development. This suggests that the major role of MeCWINVs was in the regulation of carbon exportation from source leaves, and the sucrose to hexose ratio in the apoplast. The role of these enzymes in the sucrose unloading to tuber was less significant.

## Supplementary Information



## Figures and Tables

**Table 1.** Basic information of the six cassava cell wall invertase genes (*MeCWINVs*).

Genes	Accession numbers	Genomic location	ORF length	Length (a.a)
*MeCWINV1*	JQ339929	scaffold00206: 207290–10385	1779	592
*MeCWINV2*	JX291160	scaffold06550: 791589–793834	1728	575
*MeCWINV3*	JN801147	scaffold12525: 531768–539616	1731	576
*MeCWINV4*	JQ792172	scaffold05875: 522938–525387	1731	576
*MeCWINV5*	JX291159	scaffold06550: 798389–800908	1731	576
*MeCWINV6*	JQ339930	scaffold02892: 67903–72504	1728	575

**Table 2.** Gene specific primers of *MeCWINVs* used for RT-PCR amplification.

Gene	Forward primer (5′ to 3′)	Reverse primer (5′ to 3′)
*MeCWINV1*	CGCGGATCCAAAATAGAGATGGAAGCAAT	CGCGTCGACATCATTTCTCAAACATACCC
*MeCWINV2*	CATGCCATGGTCTTCTTCATCTTCAGTC	CGGGTTACCTTTTATCTTTAGCTCAGC
*MeCWINV3*	TCAATCAAAGGAGCTATGGACA	GCACTGGCTTCTTTTATTTCATC
*MeCWINV4*	TATGGATCCCTCACCAGCATGGCTATCA	CATGTCGACTGTCAACCCTGGCTATTTCTCA
*MeCWINV5*	TCATCCCGCAAATTCAACATT	GCTTACTTCTTTTCCACCTTATTTTTT
*MeCWINV6*	CTCATTATCCCAAACAGATCAACC	ATTCTCACTTCTTTAAGCAGTCTCA

**Table 3.** Primers for *MeCWINVs* used for qRT-PCR amplification.

Gene	Forward primer (5′ to 3′)	Reverse primer (5′ to 3′)
*MeCWINV1*	CAATGGAACTCAGAGCATAACC	TCTCAAACATACCCACAAAACA
*MeCWINV2*	ATAGGAGGATTTTGTGGGGTTG	TGACTTGTTTTCTACTGGCATCT
*MeCWINV3*	GCTTCGTGTGAATCCAGTCC	TCTGCCTGTGATGCTGTGA
*MeCWINV4*	ATTTACTGTGAAGGAAGATGCG	TCTGTAGTTGTCAACCCTGGCT
*MeCWINV5*	TTGGTAAGGCTCACTTGTTTGTAT	CAAGTATGGACATGTTAGACAGAATG
*MeCWINV6*	GGAAGATTGTGGTGGGGAGTAG	TGCCCAACAACTGAAGTATCC

## Abbreviations

a.a: amino acids
bp: base pair
BLAST: basic local alignment search tool
ORF: open reading frame
RACE: rapid amplification of cDNA ends
RT-PCR: reverse transcription polymerase chain reaction
qRT-PCR: real-time quantitative PCR
