# Supplementary material for: Genome-Wide Identification, 3D Modeling, Expression and Enzymatic Activity Analysis of Cell Wall Invertase Gene Family from Cassava (Manihot esculenta Crantz)
Source: Int J Mol Sci. 2014 Apr 28;15(5):7313–31. doi: 10.3390/ijms15057313 (PMC4057674; doi:10.3390/ijms15057313)
Supplement: Supplementary file 1 [file ijms-15-07313-s001.pdf]

Supplementary Information

Table S1. Identity analysis of the six cell wall invertase coding sequences and their predicted amino acid in cassava.

**Amino acid identity**	**Nucleotides identity**					
	MeCWINV1	***MeCWINV2***	***MeCWINV3***	***MeCWINV4***	***MeCWINV5***	***MeCWINV6***
MeCWINV1	-	56.90%	59.86%	60.00%	57.41%	57.82%
MeCWINV2	48.25%	-	60.64%	59.01%	86.77%	81.70%
MeCWINV3	53.16%	57.80%	-	82.96%	61.19%	59.83%
MeCWINV4	52.65%	53.60%	77.93%	-	60.10%	58.50%
MeCWINV5	50.83%	80.38%	57.98%	54.42%	-	79.47%
MeCWINV6	50.58%	77.78%	55.31%	51.11%	74.70%	-

© 2014 by the authors; licensee MDPI, Basel, Switzerland. This article is an open access article distributed under the terms and conditions of the Creative Commons Attribution license (http://creativecommons.org/licenses/by/3.0/).
